# Pharmacological strategies for intestinal ischemia–reperfusion injury: mechanisms and therapeutic advances

**DOI:** 10.3389/fphar.2026.1892535

**Published:** 2026-09-03

**Authors:** Wenhui Wang, Jingran Ma, Yiru Wen, Jieting Liu

**Affiliations:** 1 The Second Hospital and Clinical Medical School, Lanzhou University, Lanzhou, China; 2 Department of Anesthesiology, The Second Hospital and Clinical Medical School, Lanzhou University, Lanzhou, China

**Keywords:** cell death, drug delivery systems, IIRI, inflammatory responses, oxidative stress

## Abstract

Intestinal ischemia–reperfusion injury is a life-threatening complication of mesenteric ischemia, shock, major surgery, and transplantation. Reperfusion triggers oxidative stress, inflammation, barrier failure, microcirculatory dysfunction, and regulated cell death, leading to mucosal and remote-organ injury. This narrative review summarizes pharmacological strategies targeting oxidative injury, excessive inflammation, dysregulated cell death, and drug delivery, while considering experimental models, administration protocols, and translational feasibility. Thiol compounds, polyphenols, melatonin, SIRT-axis modulators, anti-inflammatory agents, ferroptosis and autophagy modulators, and targeted delivery platforms show therapeutic potential; however, most evidence derives from heterogeneous animal models using prophylactic or peri-reperfusion dosing, and clinical evidence remains very limited. Future studies should use clinically realistic treatment windows, standardized models and outcomes, and rational multimodal regimens.

## 1 Introduction

**FIGURE 1:**
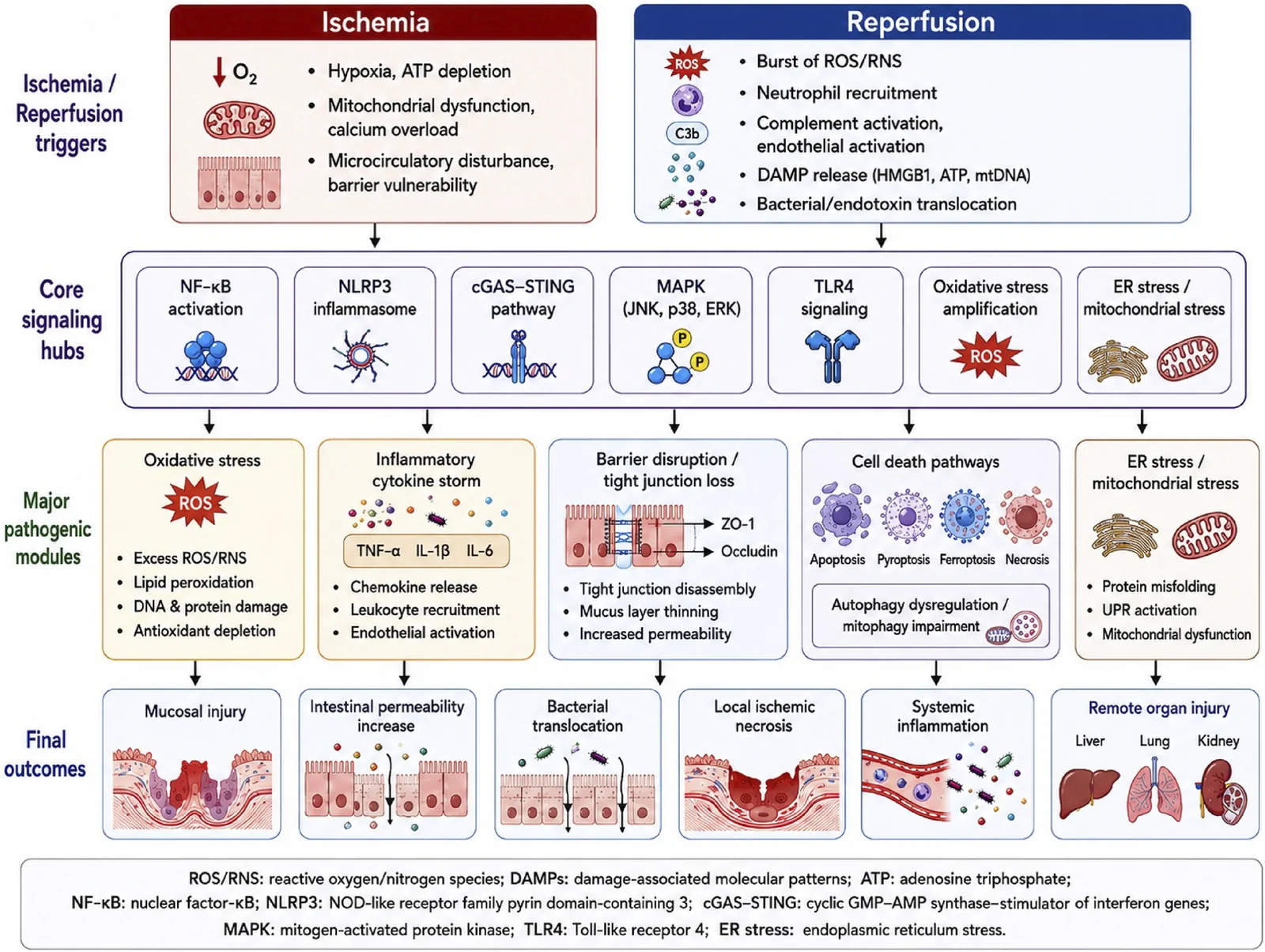
Pathophysiological cascade and major outcomes of intestinal ischemia–reperfusion injury. Ischemia-induced hypoxia and cellular stress are amplified during reperfusion through oxidative, inflammatory, barrier-disruptive, and regulated cell-death pathways, culminating in mucosal injury, bacterial translocation, systemic inflammation, and remote-organ damage. Abbreviations: ATP, adenosine triphosphate; C3b, complement component 3b; cGAS–STING, cyclic GMP–AMP synthase–stimulator of interferon genes; DAMPs, damage-associated molecular patterns; ER, endoplasmic reticulum; HMGB1, high-mobility group box 1; IL, interleukin; MAPK, mitogen-activated protein kinase; mtDNA, mitochondrial DNA; NF-κB, nuclear factor kappa B; NLRP3, NOD-like receptor family pyrin domain-containing 3; RNS, reactive nitrogen species; ROS, reactive oxygen species; TLR4, Toll-like receptor 4; TNF-α, tumor necrosis factor alpha; UPR, unfolded protein response; ZO-1, zonula occludens 1.

Intestinal ischemia–reperfusion injury (IIRI) is a frequent and devastating perioperative complication associated with intestinal obstruction, hemorrhagic shock, cardiac surgery, transplantation, and other critical-care settings (Zhong et al., 2025). Restoration of intestinal perfusion is essential; however, reperfusion can aggravate tissue injury and cannot fully interrupt the pathophysiological cascade initiated by ischemia. Oxidative stress, dysregulated inflammatory signaling, microcirculatory dysfunction, and regulated cell death reinforce one another to form a self-amplifying cycle (Zhong et al., 2025; Archontakis-Barakakis et al., 2025), as summarized in Figure 1. Complement activation is considered within this broader inflammatory network rather than as a separate pharmacological category because intestine-specific evidence for complement-directed therapy remains limited to preclinical studies (Wada et al., 2001). In recent years, numerous pharmacological interventions targeting these interconnected mechanisms have been investigated to alleviate IIRI.

To identify relevant evidence, PubMed, Web of Science, and Embase were searched for English-language publications from 1 January 2000, to 31 March 2026. Search terms combined IIRI with oxidative stress, inflammation, regulated cell death, antioxidants, anti-inflammatory agents, nanoparticles, exosomes, and liposomes, together with representative drugs including N-acetylcysteine, mesna, cysteamine, resveratrol, melatonin, metformin, dexmedetomidine, and rapamycin. Studies were included if they directly examined IIRI or related intestinal hypoperfusion/reperfusion models, evaluated a pharmacological or drug-delivery intervention, and reported mechanistic, inflammatory, barrier-related, histological, or survival outcomes. Duplicates, non-intestinal or non-pharmacological studies, inaccessible full texts, and reports with insufficient mechanistic or translational information were excluded. The heterogeneous evidence was synthesized narratively.

**FIGURE 2:**
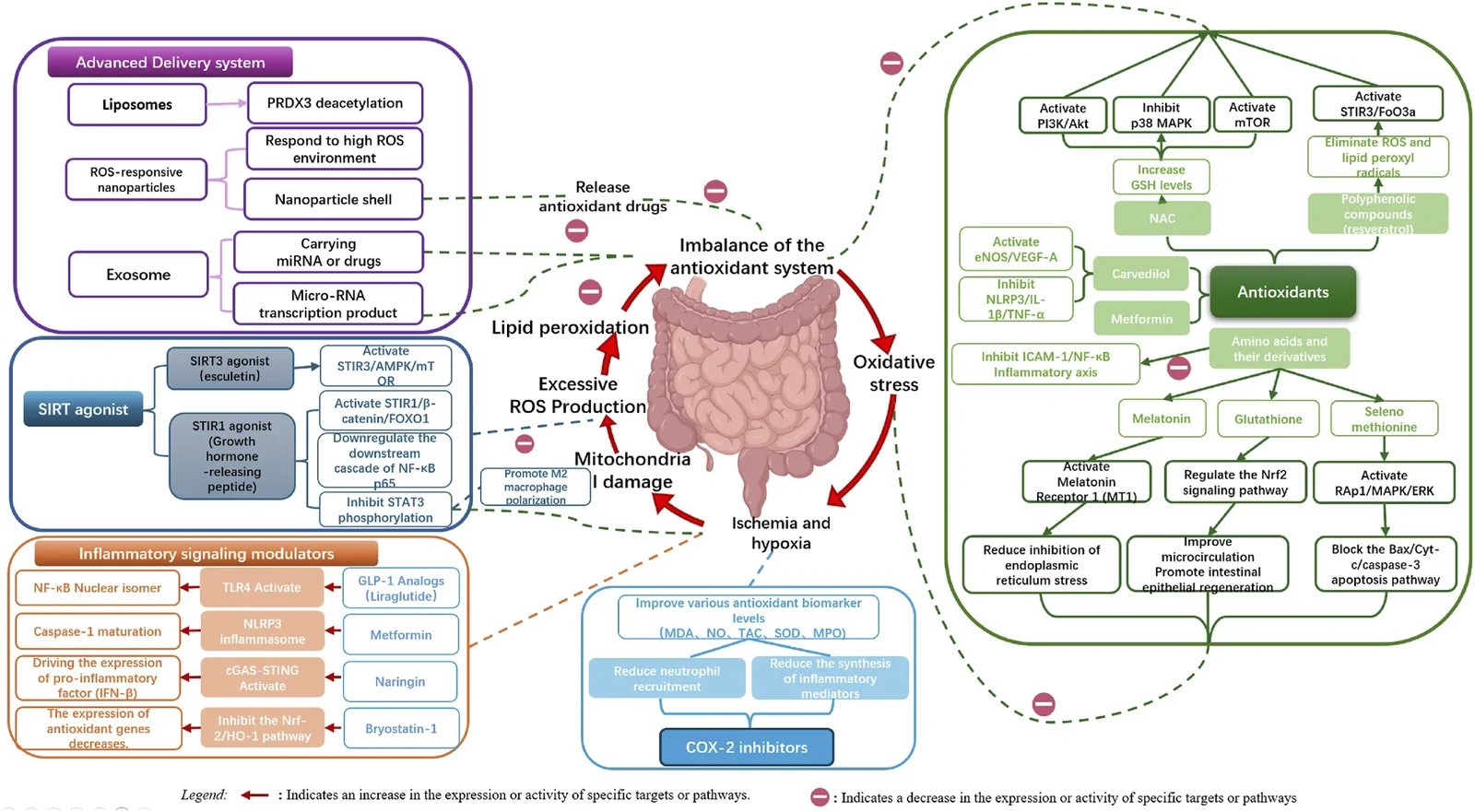
Pharmacological strategies and molecular targets in intestinal ischemia–reperfusion injury. Representative antioxidants, sirtuin agonists, inflammatory-signaling modulators, cyclooxygenase-2 inhibitors, and advanced delivery systems are mapped to their principal targets within the oxidative stress–lipid peroxidation–mitochondrial injury cycle. Red arrows indicate increased expression or activity, whereas minus symbols indicate decreased expression or activity. Abbreviations: AMPK, AMP-activated protein kinase; Bax, Bcl-2-associated X protein; cGAS–STING, cyclic GMP–AMP synthase–stimulator of interferon genes; COX-2, cyclooxygenase-2; Cyt-c, cytochrome c; eNOS, endothelial nitric oxide synthase; ERK, extracellular signal-regulated kinase; FoxO3a, forkhead box O3a; GLP-1, glucagon-like peptide 1; GSH, glutathione; HO-1, heme oxygenase 1; ICAM-1, intercellular adhesion molecule 1; IFN-β, interferon beta; IL-1β, interleukin 1 beta; MAPK, mitogen-activated protein kinase; MDA, malondialdehyde; miRNA, microRNA; MPO, myeloperoxidase; MT1, melatonin receptor 1; mTOR, mechanistic target of rapamycin; NAC, N-acetylcysteine; NF-κB, nuclear factor kappa B; NLRP3, NOD-like receptor family pyrin domain-containing 3; NO, nitric oxide; Nrf2, nuclear factor erythroid 2-related factor 2; PI3K/Akt, phosphoinositide 3-kinase/protein kinase B; PRDX3, peroxiredoxin 3; Rap1, Ras-related protein 1; ROS, reactive oxygen species; SIRT, sirtuin; SOD, superoxide dismutase; STAT3, signal transducer and activator of transcription 3; TAC, total antioxidant capacity; TLR4, Toll-like receptor 4; TNF-α, tumor necrosis factor alpha; VEGF-A, vascular endothelial growth factor A.

Based on the retrieved literature, the structure of this narrative review is outlined below. Section 2 categorizes pharmacological interventions by their core targeted pathophysiological cascades, covering oxidative stress, exaggerated inflammatory responses, and dysregulated cell death. Section 3 describes innovative targeted drug delivery systems, whereas Section 4 compares the therapeutic prospects of representative drugs and identifies key barriers to clinical translation. Section 5 summarizes future research perspectives and the inherent limitations of this review. By systematically integrating molecular mechanisms, preclinical experimental models, standardized administration regimens, and translational challenges, this review seeks to establish a unified theoretical framework to facilitate the screening and translational development of pharmacotherapies for IIRI. Figure 2 and Table 1 summarize the primary pharmacological intervention targets and representative experimental administration protocols.

**TABLE 1:** Representative pharmacological interventions and experimental protocols for IIRI.

Therapeutic class	Specific drug	Mechanism of action	Key signaling pathway/target	Model and administration	References
Thiol Compounds	N-acetylcysteine (NAC)	↑GSH, ↓MDA, ↑SOD, anti-apoptosis, anti-inflammation	p38 MAPK, PI3K/Akt/mTOR, TLR4/NF-κB	Rat SMAO (60 min/2–4 h) Intraperitoneal NAC before ischemia, with optional repeat dosing before reperfusion	(Hou et al., 2015; Xu et al., 2014; Leite et al., 2021; Kalimeris et al., 2016; Uraz et al., 2013; Liu et al., 2017)
Polyphenolic Compounds	Resveratrol	ROS scavenging, ↑SOD2/CAT, inhibits ferroptosis	SIRT3/FoxO3a, ↓ACSL4, ↑GPX4	Rat SMAO (45 min/7 days)Resveratrol 10 mg/kg before and/or after I/R	(Bertoni et al., 2018; Xu et al., 2025; Borges et al., 2016)
Polyphenolic Compounds	Quercetin	↑IL-10, ↓MPO/COX-2, promotes mucosal repair	CD68^+^ macrophage activation; anti-inflammatory effect	Rat SMAO (60 min/4–24 h) Intraperitoneal quercetin 30 min before ischemia	(Curgal et al., 2018; Tóth et al., 2017)
	Emodin	↑HO-1, anti-oxidative, anti-apoptotic	PI3K/Akt/HO-1	Mouse SMAO and Caco-2 OGD/ROral emodin for 5 days before I/R; post-OGD/R treatment *in vitro*	(Liu et al., 2024a; Chen et al., 2024)
	Baicalin	Inhibits ferroptosis *via* Nrf2/GPX4 pathway	Nrf2/GPX4	Rat intestinal I/R and cellular OGD/R Baicalin treatment; route and timing not specified	(Zhai and Liu, 2024)
	Corilagin	Inhibits ERS, restores autophagy, antioxidant	BiP/UPR, AMPK/Sirt1	Rat/mouse intestinal I/R and Caco-2 H/R Corilagin pretreatment	(Wang et al., 2024; Li et al., 2023)
	Corilagin	Inhibits NLRP3-mediated pyroptosis	NLRP3 ↓, apoptosis ↓	Mouse intestinal I/R Corilagin pretreatment with intestinal and lung outcomes	(Li et al., 2022)
	*Fucus vesiculosus* extract	↓MDA/MPO/IL-1β, ↑CAT/IL-10	Antioxidant and anti-inflammatory	Rat SMAO (60 min/3–24 h) Treatment 24 h before ischemia, at ischemia onset, or at reperfusion onset	(Sánchez-Bonet et al., 2025)
Amino Acids and Their Derivatives	Melatonin	↓ER stress, ↑SOD/GPx, ↓NF-κB, anti-apoptosis	MT1, Nrf2/HO-1, TLR4/MyD88/NF-κB	Rat SMAO (30–60 min)Intraperitoneal melatonin before I/R; acute or 7-day assessment	(Tas et al., 2015; Missawi et al., 2023; Liu et al., 2022a; Yang et al., 2020a; Yin et al., 2024; Özkan et al., 2018)
	Glutathione (GSH)	ROS scavenging, maintains membrane integrity	Nrf2 pathway	Mechanistic or review evidence Specific intervention protocol not reported	(Bhattacharyya et al., 2014; Ngo and Duennwald, 2022)
	S-nitrosoglutathione (GSNO)	↓MDA/MPO, inhibits NF-κB and iNOS	NF-κB, anti-inflammatory	Rat SMAO (30 min/180 min) Intravenous GSNO 0.25 mg/kg before reperfusion	(Turan et al., 2018)
	Selenomethionine	↑GPx/CAT/SOD, inhibits apoptosis	Bax/Cyt-c/caspase-3, Rap1/MAPK/ERK	Mouse intestinal I/R SeMet pretreatment; route not specified	(Liu et al., 2020; Liu et al., 2022b; Wan et al., 2020)
SIRT activator	Ghrelin	Activates SIRT1/FoxO1, anti-oxidative and anti-apoptotic	GHSR-1α/SIRT1/FoxO1	Mouse SMAO and Caco-2 H/RIntraperitoneal ghrelin 50 μg/kg 20 min before I/R	(Liao et al., 2024)
SIRT activator	SRT1720	Improves organ damage post-I/R	SIRT1	Mouse SMAO (45 min/4 h) Intravenous SRT1720 at reperfusion	(Hansen et al., 2016)
	Vinpocetine	Promotes M2 polarization, anti-inflammatory	SIRT1/SOCS3/STAT3	Rat intestinal I/R (60 min/2 h)Oral 20 mg/kg/d for 2 weeks before I/R	(Elwany et al., 2023)
	SIRT3 activator (endogenous)	Deacetylates PRDX3, ↑H_2_O_2_ scavenging	SIRT3/PRDX3	Mouse SMAO and Caco-2 H/R Genetic or transfection-based SIRT3/PRDX3 intervention	(Wang et al., 2020)
	Esculetin	Activates SIRT3/AMPK/mTOR, induces autophagy	SIRT3/AMPK/mTOR	Rat intestinal I/R and IEC-6 H/R Esculetin 10 or 25 mg/kg *in vivo*	(Shen et al., 2023)
α_1_- and β-adrenergic receptor blocker	Carvedilol	Anti-oxidative, anti-inflammation, anti-apoptosis	eNOS/VEGF-A ↑, NLRP3/IL-1β/TNF-α ↓	Rat intestinal I/R Carvedilol 10 or 20 mg/kg; route and timing not specified	(Refaie et al., 2025)
COX-2 inhibitor	Parecoxib sodium	↓COX-2, ↓IL-1β/IL-6/ICAM-1, anti-apoptosis	COX-2 ↓, caspase-3 ↓, Bcl-2 ↑	Rat SMAO (30 min/1 h)COX-2 inhibitor plus vitamin C before and after I/R	(Gugliandolo et al., 2020)
COX-2 inhibitor	Firocoxib	Synergistic antioxidant and anti-inflammatory	COX-2/PGs/NF-κB	Rat SMAO (30 min/1 h)Firocoxib plus vitamin C before and after I/R	(Gugliandolo et al., 2020)
COX-2 inhibitor	Celecoxib	↓IL-1β/MPO, preserves tight junctions	COX-2, Claudin-1/Occludin	Rat mesenteric I/ROral gavage for 8 days; last dose 2 h before I/R	(László et al., 2024)
NF-κB Signaling Pathway Inhibitors	Liraglutide	Inhibits NF-κB nuclear translocation, activates PI3K/Akt	NF-κB ↓, PI3K/Akt ↑	Transient mouse SMAO Dose-escalation liraglutide pretreatment	(Zou and Wang, 2022)
	Lixisenatide	Inhibits IκB-α phosphorylation, ↓TNF-α/IL-1β	NF-κB ↓ (IκB-α dependent)	Rat cerebral I/R Lixisenatide pretreatment	(Gad et al., 2020)
	Dexmedetomidine	Inhibits TLR4/MyD88/NF-κB, ↓Nupr1, ↑autophagy	TLR4/MyD88/NF-κB ↓, Nupr1 ↓	Rat SMAO and Caco-2 OGD/R Dexmedetomidine pretreatment	(Yang et al., 2021; Wu et al., 2024)
NLRP3 Inflammasome Pathway Inhibitors	Metformin	Inhibits TXNIP-NLRP3-GSDMD pyroptosis axis	NLRP3 ↓, GSDMD ↓, caspase-1 ↓	Mouse SMAO and Caco-2 OGD/RMetformin pretreatment before I/R (100 or 200 mg/kg)	(Jia et al., 2020)
	Metformin	↓MDA/MPO/ICAM-1/NF-κB, ↑GSH	ICAM-1/NF-κB axis	Rat SMAOOral gavage 50–200 mg/kg/d for 1 week before I/R	(Turan et al., 2025)
	Honokiol	Inhibits NLRP3 inflammasome, ↑SIRT3	SIRT3/NLRP3/caspase-1/IL-1β ↓	Rat SMA occlusion and IEC-6 H/R Honokiol 10 mg/kg *in vivo*	(Park et al., 2020; Wu et al., 2025)
cGAS-STING pathway inhibitors	Naringin	Blocks STING phosphorylation, ↓inflammation	cGAS-STING ↓	Rat intestinal I/R and cellular H/R Naringin treatment	(Gu et al., 2023)
	Ginsenoside Rg3	Modulates gut flora → GPR43 → cGAS-STING ↓	Microbiota-GPR43-cGAS-STING	Enteric viral-infection model Ginsenoside Rg3 treatment	(Wang et al., 2023b)
Nrf2/HO-1 pathway activator	Methane-rich saline	Activates Nrf2/HO-1, inhibits ferroptosis	Nrf2/HO-1 ↑	Mouse SMAO and Caco-2 OGD/R Methane-rich saline treatment	(Fan et al., 2024)
	Bryostatin-1	Activates Nrf2/HO-1, preserves barrier function	Nrf2/HO-1 ↑	Mouse SMAO (60 min/120 min) Intraperitoneal bryostatin-1 during the experiment	(Liu et al., 2023)
	Dimethyl fumarate	Activates Nrf2/HO-1/Bcl-2, inhibits GSK-3β	Nrf2/HO-1 ↑, Wnt/β-catenin ↑	Rat intestinal I/R (60 min/6 h)Oral 15 or 25 mg/kg/d for 14 days before I/R	(Gendy et al., 2021)
	APG	Inhibits ferroptosis, ↓Fe^2+^/ROS/MAO-B	HO-1 ↓, MAO-B ↓	Mouse intestinal I/R Intraperitoneal APG 2–8 mg/kg 15 min after ischemia onset	(Feng et al., 2022)
	Rapamycin	Enhances autophagy, ↓NF-κB, anti-inflammatory	mTOR ↓, LC3-II ↑, NF-κB ↓	Ulcerative-colitis model Rapamycin treatment	(Xu et al., 2024a)
Novel Drug Delivery Systems	Resveratrol-loaded nanogel	Targeted delivery, ↑bioavailability 4.7×	ROS-responsive release	*In vitro* gastrointestinal release and **C*. elegans* model Resveratrol nanogel treatment	(Xu et al., 2024b)
	Rapamycin-MnO_2_@MM	↓ROS, ↑autophagy, tissue-targeted	ROS scavenging, autophagy ↑	Mouse SMAO (60 min/2 h) Intravenous nanoparticle treatment	(Sheng et al., 2025)
	Apigenin-7-glucoside NP	Inhibits ferroptosis *via* ATF3/SLC7A11	ATF3/SLC7A11-mediated ferroptosis ↓	Mouse intestinal I/R and cellular models Apigenin-7-glucoside nanoparticle treatment	(Zhao et al., 2024)
	miR-4334/219/338 (milk-derived)	Inhibits apoptosis and inflammation	TLR4/NF-κB ↓, p53 ↓	LPS intestinal-injury and epithelial-cell models Milk exosome or miRNA transfection	(Alzhrani et al., 2021; Xie et al., 2019)
	circEZH2_005	Promotes Lgr5^+^ stem-cell proliferation	circEZH2_005/hnRNPA1/*Gprc5a*	Mouse intestinal I/R (1 h/2 h) and organoid H/R Adenoviral circEZH2_005 overexpression	(Zhang et al., 2023)
	miR-144-3p-exosome	Regulates oxidative stress and inflammation	PTEN/Akt/Nrf2 ↑, TLR4/NF-κB ↓	Mouse intestinal I/R and IEC-6 OGD/R BMSC-exosome treatment	(Zhang et al., 2022; Sheng et al., 2021)
	Human breast milk-derived exosomes	↓TNF-α, alleviates intestinal damage	Anti-inflammatory	Neonatal rat SMAO (30 min/6 h) Oral human breast-milk-derived exosomes	(Wang et al., 2022)

Abbreviations: ACSL4, acyl-CoA, synthetase long-chain family member 4; Akt, protein kinase B; AMPK, AMP-activated protein kinase; APG, apigenin-7-O-β-D-(6″-p-coumaroyl)-glucopyranoside; ATF3, activating transcription factor 3; Bax, Bcl-2-associated X protein; Bcl-2, B-cell lymphoma 2; BiP, binding immunoglobulin protein; BMSC, bone marrow mesenchymal stem cell; CAT, catalase; cGAS, cyclic GMP–AMP, synthase; circEZH2_005, circular RNA EZH2_005; COX-2, cyclooxygenase-2; Cyt-c, cytochrome c; eNOS, endothelial nitric oxide synthase; ERK, extracellular signal-regulated kinase; ERS, endoplasmic reticulum stress; FOXO1/FoxO1 and FoxO3a, forkhead box O1 and O3a; GHSR-1α, growth hormone secretagogue receptor 1α; GPx/GPX4, glutathione peroxidase/glutathione peroxidase 4; GPR43, G protein-coupled receptor 43; *Gprc5a*, G protein-coupled receptor class C group 5 member A; GSDMD, gasdermin D; GSH, glutathione; GSK-3β, glycogen synthase kinase 3 beta; GSNO, S-nitrosoglutathione; H/R, hypoxia/reoxygenation; hnRNPA1, heterogeneous nuclear ribonucleoprotein A1; HO-1, heme oxygenase 1; ICAM-1, intercellular adhesion molecule 1; IEC-6, rat intestinal epithelial cell line 6; IL, interleukin; iNOS, inducible nitric oxide synthase; I/R, ischemia/reperfusion; IκB-α, inhibitor of nuclear factor kappa B alpha; LC3-II, microtubule-associated protein 1 light chain 3-II; Lgr5, leucine-rich repeat-containing G-protein-coupled receptor 5; LPS, lipopolysaccharide; MAO-B, monoamine oxidase B; MAPK, mitogen-activated protein kinase; MDA, malondialdehyde; miR/miRNA, microRNA; MnO_2_@MM, macrophage-membrane-coated manganese dioxide nanoparticles; MPO, myeloperoxidase; MT1, melatonin receptor 1; mTOR, mechanistic target of rapamycin; MyD88, myeloid differentiation primary response 88; NAC, N-acetylcysteine; NF-κB, nuclear factor kappa B; NLRP3, NOD-like receptor family pyrin domain-containing 3; NP, nanoparticle; Nrf2, nuclear factor erythroid 2-related factor 2; Nupr1, nuclear protein 1; OGD/R, oxygen–glucose deprivation/reoxygenation; PGs, prostaglandins; PI3K, phosphoinositide 3-kinase; PRDX3, peroxiredoxin 3; PTEN, phosphatase and tensin homolog; Rap1, Ras-related protein 1; ROS, reactive oxygen species; SeMet, selenomethionine; SIRT, sirtuin; SLC7A11, solute carrier family 7 member 11; SMA/SMAO, superior mesenteric artery/superior mesenteric artery occlusion; SOCS3, suppressor of cytokine signaling 3; SOD/SOD2, superoxide dismutase/superoxide dismutase 2; STAT3, signal transducer and activator of transcription 3; STING, stimulator of interferon genes; TLR4, Toll-like receptor 4; TNF-α, tumor necrosis factor alpha; TXNIP, thioredoxin-interacting protein; UPR, unfolded protein response; VEGF-A, vascular endothelial growth factor A; Wnt, Wingless-related integration site; ZO-1, zonula occludens 1.

## 2 Pharmacological strategies targeting major pathophysiological processes

### 2.1 Antioxidant strategies

#### 2.1.1 Thiol compounds

Thiol compounds buffer oxidative stress through reversible thiol–disulfide exchange and replenishment of intracellular reduced glutathione (GSH). These actions help preserve protein and non-protein thiol pools, limit lipid peroxidation, and maintain the mucosal redox environment during reperfusion.

The representative drug N-acetylcysteine (NAC) can penetrate cell membranes and directly increase intracellular levels of reduced glutathione (GSH) (Hou et al., 2015). It also improves the intestinal microbiota by increasing the abundance of lactic acid bacteria and *Bifidobacterium* while decreasing the abundance of *E. coli* (*Escherichia coli*) (Xu et al., 2014). Studies have shown that NAC pretreatment can significantly reduce malondialdehyde (MDA) levels in rats with IIRI, increase the activity of superoxide dismutase (SOD), and alleviate apoptosis by inhibiting the phosphorylation of p38 mitogen-activated protein kinase (p38 MAPK) (Leite et al., 2021). In addition, NAC reduces serum lipid peroxidation and mitigates liver injury after IIRI. Kalimeris et al. (2016) found that NAC increased glutathione peroxidase-1 (GPx-1) activity in the lung tissues of Wistar rats. In addition to restoring antioxidant enzyme activity, NAC can also downregulate pro-inflammatory cytokines such as tumor necrosis factor-α (TNF-α), interleukin-1β (IL-1β), and IL-6 (Uraz et al., 2013). Further studies have confirmed that NAC exerts antioxidant and anti-inflammatory effects by activating multiple signaling pathways, including phosphoinositide 3-kinase/protein kinase B (PI3K/Akt)/mechanistic target of rapamycin (mTOR), EGFR, Toll-like receptor 4 (TLR4)/nuclear factor kappa-B (NF-κB), AMPK, and type I interferon (type I IFN), thereby improving intestinal injury (Liu et al., 2017).

Beyond NAC, other thiol-active agents further support the relevance of redox-buffering strategies in IIRI. In a rat intestinal ischemia/reperfusion model, mesna reduced oxidative stress, preserved the mucosal GSH/GSSG ratio, and attenuated histological injury and apoptosis, suggesting that maintenance of glutathione redox balance is itself a mechanistically meaningful protective axis during reperfusion (Ypsilantis et al., 2006). More recently, cysteamine was shown to restore GSH, GPx, and both protein and non-protein thiol pools while reducing H_2_O_2_, MDA, nitrite, MPO, TNF-α, and IL-6 in experimental intestinal reperfusion injury, indicating that reinforcement of intracellular thiol activity and thiol-disulfide homeostasis may broaden the therapeutic scope of thiol compounds beyond NAC alone (Alabi et al., 2023). These observations shift attention from single-agent antioxidant effects toward a broader thiol-redox framework involving GSH/GSSG balance, cysteine-related buffering, and preservation of the intestinal mucosal redox environment.

#### 2.1.2 Polyphenolic compounds

Polyphenolic compounds discussed in IIRI include resveratrol, quercetin, emodin, baicalin, corilagin, and phlorotannin-rich extracts. Their protective actions extend beyond direct radical scavenging and include metal chelation, suppression of ROS/RNS generation, preservation of endogenous antioxidant enzymes, and modulation of ferroptosis, autophagy, and inflammatory signaling.

Resveratrol, a stilbene polyphenol abundant in grapes, berries, and red wine, exerts direct free radical scavenging effects through its phenolic hydroxyl groups, activates the SIRT3/FoxO3a pathway, upregulates the expression of SOD2 and catalase, inhibits ROS generation, and thereby reduces lipid peroxidation and ferroptosis (Bertoni et al., 2018). Xu et al. (2025) reported that resveratrol decreases the Fe^2+^ level and the protein expression of acyl-CoA synthetase long-chain family member 4 (ACSL4), while increasing the levels of GSH and GPX4 in mice with IIRI, thereby alleviating intestinal injury by inhibiting ferroptosis. Borges et al. (2016) confirmed that preventive intravenous injection of resveratrol protects the myenteric plexus of the ileum in Wistar rats and inhibits the proliferation of glial cells after I/R.

Quercetin is widely present in tea, fruits, and vegetables. Curgal et al. (2018) found that quercetin pretreatment increases the level of the anti-inflammatory cytokine IL-10 in the serum and jejunal tissue of mice with I/R, promotes intestinal gland elongation, increases the number of CD68^+^ cells in the lamina propria, and accelerates jejunal mucosal repair. Tóth et al. (2017) further confirmed that quercetin reduces the activity of myeloperoxidase (MPO) by inhibiting neutrophil infiltration and decreases the expression of COX-2, thereby alleviating intestinal mucosal injury.

Emodin is a plant-derived anthraquinone with antioxidant and anti-inflammatory activity. Liu Y. et al. (2024) demonstrated in a mouse IIRI model and a Caco-2 oxygen-glucose deprivation/reoxygenation model that emodin protected intestinal tissue through Akt-mediated HO-1 upregulation.

Baicalin, a flavonoid derived mainly from *Scutellaria baicalensis* Georgi, has shown antioxidant and anti-inflammatory activity in ischemic injury models. Zhai and Liu (2024) reported that baicalin attenuated oxidative stress and inflammation by activating Nrf2/GPX4 signaling and suppressing ferroptosis in IIRI.

Natural antioxidants derived from marine and botanical sources have received increasing attention in IIRI research. Because autophagy regulation and inflammasome activation are closely intertwined in inflammatory injury, multitarget natural products that modulate both processes are of particular interest (Zhao et al., 2021). Among them, corilagin (Cor), a water-soluble ellagitannin identified in several medicinal plants, has emerged as a representative multitarget natural product with antioxidant, anti-inflammatory, and barrier-protective properties. Histological and immunohistochemical studies showed that corilagin reduced intestinal 4-HNE and IL-1β expression while restoring the tight-junction proteins Occludin and ZO-1 in murine intestinal tissue (Wang Y. et al., 2023). Mechanistically, Wang et al. (2024) demonstrated that corilagin directly interacts with BiP/GRP78, promotes its ubiquitin-dependent degradation, and suppresses excessive activation of the unfolded protein response branches IRE1, PERK, and ATF6, thereby alleviating oxidative stress, apoptosis, and tissue injury in IIRI. Li et al. (2023) further showed that corilagin restores autophagic activity through the AMPK/Sirt1 pathway, promotes p62 degradation, and increases Beclin1 and LC3II expression, thus improving intestinal barrier dysfunction. In parallel, corilagin has also been reported to attenuate concomitant lung injury after intestinal ischemia–reperfusion by inhibiting NLRP3 inflammasome activation and pyroptosis (Li et al., 2022). Taken together, these findings suggest that corilagin is not merely an antioxidant scavenger, but a pleiotropic natural compound capable of simultaneously modulating ER stress, autophagy, inflammasome activation, and barrier integrity.

*Fucus vesiculosus* is rich in phlorotannins, a class of polyphenolic compounds. In recent years, phlorotannins have been shown to possess potent antioxidant and anti-inflammatory activities. Sánchez-Bonet et al. (2025) found in a rat model of IIRI that *Fucus vesiculosus* extract significantly reduced the levels of MDA, MPO, and IL-1β, restored catalase (CAT) activity and IL-10 expression, improved intestinal villus architecture, and alleviated tissue damage. These findings indicate that algal polyphenols may protect against IIRI by enhancing endogenous antioxidant defenses and inhibiting inflammatory mediator release, while also providing a new rationale for the development of marine-derived antioxidant functional foods or adjunctive agents.

#### 2.1.3 Amino acids and their derivatives

Melatonin (N-acetyl-5-methoxytryptamine), an indoleamine derived from tryptophan metabolism, exhibits robust antioxidant and anti-inflammatory activity and remains one of the most extensively studied cytoprotective agents in IIRI. Experimental studies indicate that melatonin attenuates intestinal injury by reducing ER stress through melatonin receptor 1 (MT1), lowering MDA levels, increasing SOD and GPx activity, and limiting intestinal epithelial apoptosis (Tas et al., 2015). In addition, melatonin suppresses NF-κB activation, reduces TNF-α and IL-6 release, and enhances endogenous antioxidant defense, thereby dampening the inflammatory amplification that accompanies reperfusion (Missawi et al., 2023). Melatonin may also activate the Nrf2/HO-1 axis and inhibit inflammasome-related signaling through TLR4/MyD88, further strengthening epithelial resilience against oxidative injury (Liu S. et al., 2022). Beyond local intestinal protection, Yang B. et al. (2020) reported that melatonin alleviated neuroinflammation, cognitive dysfunction, and brain injury secondary to intestinal ischemia–reperfusion in rats, supporting the concept that IIRI is a systemic rather than exclusively intestinal disorder. Additional studies have also suggested beneficial effects of melatonin on cognitive impairment and anastomotic healing in related experimental settings (Yin et al., 2024; Özkan et al., 2018). Given its pleiotropic actions, melatonin remains a clinically attractive candidate for peri-operative organ protection, although issues of timing, dose selection, and translational validation still need to be resolved.

Glutathione (GSH) is an important intracellular antioxidant that scavenges ROS through GPx. In IIRI, GSH reduces ROS generation and helps maintain cell membrane integrity, thereby alleviating oxidative damage (Bhattacharyya et al., 2014). Studies have shown that GSH pretreatment significantly decreases MDA levels in intestinal tissue, increases the activities of SOD and GPx, and attenuates IIRI. It may also enhance the expression of antioxidant enzymes through the Nrf2 signaling pathway, thereby strengthening its protective effect (Ngo and Duennwald, 2022). S-nitrosoglutathione (GSNO), an analog of glutathione, has also shown protective effects. Turan et al. (2018) found that intravenous administration of GSNO before reperfusion reduced pathological scores in the intestinal and lung tissues of rats, decreased intestinal lipid peroxidation and MPO levels, and inhibited the expression of NF-κB and inducible nitric oxide synthase (iNOS).

Selenomethionine (SeMet) is an organic selenium (Se) compound with low toxicity and high bioavailability and is often used for the treatment of selenium deficiency. SeMet may alleviate oxidative stress through the Rap1/MAPK/ERK signaling pathway and provide selenium for the synthesis of selenoproteins such as GPx, thereby helping maintain intracellular redox homeostasis and limit free-radical injury (Liu et al., 2020). Mechanistic studies have shown that SeMet can increase the levels of antioxidant enzymes such as GPx, CAT, and SOD, decrease MDA content, and enhance antioxidant capacity (Liu Z. et al., 2022). Meanwhile, it upregulates the expression of B-cell lymphoma-2 (Bcl-2), inhibits the expression of cytochrome c (Cyt-c), Bax, and caspase-3, and blocks the Bax/Cyt-c/caspase-3 apoptotic pathway, thereby significantly reducing I/R-induced apoptosis and exerting a protective effect on the intestine.

#### 2.1.4 SIRT family and mitochondrial antioxidant system

Sirtuins and mitochondrial peroxiredoxin 3 (PRDX3) form part of a coordinated nuclear–mitochondrial antioxidant network. Pharmacological or endogenous activation of SIRT1 and SIRT3 may limit IIRI by combining upstream transcriptional regulation with downstream control of mitochondrial ROS.

Silent information regulator 1 (SIRT1) is an NAD^+^-dependent deacetylase that participates in the regulation of oxidative stress, inflammation, and apoptosis. SIRT1 activators enhance SIRT1 activity and promote the deacetylation of downstream target proteins, thereby exerting both antioxidant and anti-inflammatory effects. Liao et al. (2024) demonstrated that ghrelin significantly inhibits oxidative stress and apoptosis induced by IIRI by activating the growth hormone secretagogue receptor 1α (GHSR-1α)/SIRT1/FoxO1 signaling pathway, providing a potential molecular target for intervention. Another study focused on SIRT1-specific activators and found that SRT1720 improved both local and remote organ injury after IIRI (Hansen et al., 2016). Vinpocetine pretreatment not only upregulated the expression of SIRT1 and SOCS3 but also effectively inhibited STAT3 phosphorylation, reduced the expression of pro-inflammatory factors such as IL-6, TNF-α, and IL-1β and inducible nitric oxide synthase (iNOS), and promoted the polarization of M2 anti-inflammatory macrophages, thereby improving intestinal pathological injury (Elwany et al., 2023). In summary, as an NAD^+^-dependent deacetylase, SIRT1 exerts antioxidant, anti-inflammatory, and anti-apoptotic effects by regulating transcription factors such as FoxO1, NF-κB, and STAT3.

Mitochondria are a major source of excessive ROS during reperfusion. SIRT3-mediated deacetylation enhances PRDX3 activity, promotes H_2_O_2_ clearance, and limits mitochondrial permeability transition and apoptosis. Wang et al. (2020) showed that SIRT3-dependent PRDX3 deacetylation alleviated mitochondrial oxidative damage and apoptosis in IIRI. Shen et al. (2023) further found that esculetin reduced oxidative stress and induced autophagy through the SIRT3/AMPK/mTOR pathway.

#### 2.1.5 Other antioxidant agents

Carvedilol is an α_1_-and β-adrenergic receptor blocker with additional antioxidant and anti-inflammatory properties. Refaie et al. (2025) found that carvedilol decreased MDA, increased GSH and total antioxidant capacity, activated the eNOS/VEGF-A axis, and suppressed NLRP3/IL-1β/TNF-α signaling. It also reduced caspase-3 expression and improved intestinal and cardiac histopathology, suggesting potential value for combined intestinal and remote-organ protection.

### 2.2 Pharmacological attenuation of excessive inflammatory responses

#### 2.2.1 COX-2 inhibitors

Cyclooxygenase-2 (COX-2) is an inducible enzyme whose expression is rapidly upregulated during IIRI. It catalyzes the synthesis of inflammatory mediators such as prostaglandins (PGs), exacerbating oxidative stress and tissue damage. COX-2 inhibitors reduce PG production by blocking the activity of this enzyme, thereby exerting both anti-inflammatory and indirect antioxidant effects.

COX-2 inhibition has been evaluated using agents with different selectivity profiles and treatment protocols. In a combination paradigm, firocoxib plus antioxidant therapy reduced COX-2-related mediators, cytokine release, neutrophil infiltration, and oxidative injury (Gugliandolo et al., 2020), supporting complementary modulation of prostaglandin signaling and redox stress. Nevertheless, the available studies do not justify a class-wide efficacy assumption, and agent-specific safety, selectivity, dose, and timing remain important determinants of effect.

László et al. (2024) compared the effects of two selective COX-2 inhibitors—celecoxib and rofecoxib—in a rat model of small-intestinal IIRI. The study found that celecoxib (especially at high doses) significantly reduced the levels of inflammatory factors (such as IL-1β and MPO), decreased the degradation of tight junction proteins (Claudin-1 and Occludin), and exhibited certain antioxidant and barrier-protective effects. However, it failed to significantly improve histopathological damage and might upregulate the Bax/Bcl-2 ratio, suggesting a potential pro-apoptotic risk. In contrast, rofecoxib showed no significant improvement in inflammation or tissue damage. This study indicates that although celecoxib is superior to rofecoxib in anti-inflammatory effects, its lower selectivity for COX-2 may be the key to the difference in efficacy. It also suggests that single-target inhibition of COX-2 may not be sufficient to effectively alleviate IIRI, and a multi-target intervention strategy is required.

#### 2.2.2 NF-κB signaling pathway inhibitors

The nuclear factor kappa-B (NF-κB) signaling pathway is a core mediator of the inflammatory cascade. Its activation can trigger the release of pro-inflammatory cytokines such as tumor necrosis factor-α (TNF-α) and interleukin-6 (IL-6) (Qiu et al., 2023). A variety of drugs exert protective effects against IIRI by inhibiting the NF-κB signaling pathway, making mechanistic studies of this pathway highly relevant to drug development.

Glucagon-like peptide-1 (GLP-1) analogs have shown protective effects in several pathophysiological settings. Liraglutide, a GLP-1 analog, has been found to significantly alleviate inflammation and apoptosis induced by IIRI. This protective effect is mainly mediated through two mechanisms: inhibition of NF-κB nuclear translocation to reduce downstream pro-inflammatory cytokine expression, and activation of the phosphoinositide 3-kinase/protein kinase B (PI3K/Akt) pathway, which is important for cell survival and anti-apoptosis. The synergistic action of these mechanisms improves the survival rate of mice with IIRI (Zou and Wang, 2022). Lixisenatide, a GLP-1 receptor agonist, can activate the ERK signaling pathway and reduce the production of TNF-α and IL-1β by inhibiting IκB-α phosphorylation, thereby alleviating cerebral I/R injury; a similar protective effect may also be relevant to IIRI (Gad et al., 2020).

Yang et al. further reported that dexmedetomidine (DEX) pretreatment protects rats from IIRI and improves oxygen-glucose deprivation/reoxygenation (OGD/R)-induced injury in intestinal epithelial Caco-2 cells. This cytoprotective and tissue-protective effect is associated with inhibition of the Toll-like receptor 4/myeloid differentiation factor 88 (TLR4/MyD88)/NF-κB signaling pathway, thereby reducing excessive inflammatory responses (Yang et al., 2021; Wu et al., 2024). More recent research has further shown that DEX can downregulate the expression of the stress-response protein Nupr1, enhance the autophagic activity of intestinal neurons, and improve mitochondrial homeostasis, thereby alleviating I/R-induced tissue damage and inflammation (Wu et al., 2024).

#### 2.2.3 NLRP3 inflammasome pathway inhibitors

The nucleotide-binding oligomerization domain-like receptor pyrin domain-containing 3 (NLRP3) inflammasome is an intracellular multiprotein complex that plays a central role in sterile inflammatory injury (Henedak et al., 2024). Upon sensing DAMPs and related stress signals, NLRP3 promotes caspase-1 activation, GSDMD cleavage, and the maturation and release of IL-1β and IL-18, thereby linking innate immune activation directly to pyroptotic cell death (Fu and Wu, 2023). In the setting of IIRI, this pathway is particularly important because oxidative stress, mitochondrial injury, microbial translocation, and barrier failure all converge on inflammasome activation. As a result, the NLRP3 pathway has emerged as one of the most persuasive anti-inflammatory targets in current preclinical studies of intestinal ischemia–reperfusion.

Metformin has been evaluated as a multimodal inhibitor of pyroptosis, oxidative stress, and inflammatory signaling. In experimental IIRI, pretreatment with 100 or 200 mg/kg reduced NLRP3, cleaved caspase-1, and the N-terminal fragment of gasdermin D, consistent with inhibition of the TXNIP–NLRP3–GSDMD axis (Jia et al., 2020). Turan et al. (2025) further reported lower MDA, MPO, ICAM-1, and NF-κB levels, higher GSH content, and improved histopathology, indicating concurrent suppression of neutrophil recruitment and redox injury.

Metformin also increased microbiota-derived short-chain fatty acids and GSH and improved tight-junction organization in experimental IIRI (Wang et al., 2025). These changes may limit permeability and systemic inflammatory spillover; however, the proposed microbiome-mediated mechanisms require independent validation.

Hydrogen-rich solution (HRS) treatment improves intestinal mucosal integrity (evidenced by reduced Chiu scores) and restores coagulation function in rats with IIRI. Specifically, HRS significantly lowers prothrombin time (PT), activated partial thromboplastin time (APTT), thrombin time (TT), fibrin degradation products (FDP), D-dimer, and PT-international normalized ratio (PT-INR) compared to the untreated I/R group (Yang L. et al., 2020). Mechanistically, these benefits are attributed to HRS-mediated inactivation of the NF-κB/NLRP3 pathway, which concurrently ameliorates coagulation disorders and excessive inflammation—two key drivers of I/R pathogenesis. Natural products also exhibit promising NLRP3-inhibitory activity.

Honokiol (HKL), a biphenolic neolignan isolated from *Magnolia officinalis* and other *Magnolia* species, possesses well-documented antioxidant, antibacterial, antitumor, and anti-inflammatory properties. Evidence from renal ischemia-reperfusion models has further shown that honokiol upregulates glutathione biosynthetic enzymes and exerts broad cytoprotective effects, supporting its relevance to I/R-related oxidative injury (Park et al., 2020). Wu et al. (2025) demonstrated that HKL pretreatment significantly reduces intestinal mucosal damage, lowers Chiu’s score, and decreases serum intestinal fatty acid-binding protein (i-FABP) concentration, a biomarker of intestinal epithelial injury, in I/R model rats; concurrently, HKL upregulates the expression of intestinal barrier proteins. *In vitro* studies using intestinal epithelial IEC-6 cells further confirmed that HKL reverses hypoxia/reoxygenation (H/R)-induced upregulation of NLRP3, cleaved caspase-1, IL-1β, and IL-18 (Wu et al., 2025). This reversal alleviates H/R-induced declines in IEC-6 cell viability and inhibits I/R-induced mitochondrial dysfunction and subsequent apoptosis. Notably, HKL’s protective effects may be mediated by upregulating sirtuin 3 (SIRT3), a mitochondrial deacetylase that mitigates mitochondrial dysfunction, thus targeting both the NLRP3 inflammasome and mitochondrial integrity.

#### 2.2.4 cGAS-STING pathway inhibitors

During IIRI, the release of mitochondrial DNA (mtDNA) acts as a critical damage-associated molecular pattern (DAMP) that activates the cyclic GMP-AMP synthase-stimulator of interferon genes (cGAS-STING) pathway (Zhang et al., 2020). Activation of this pathway drives the expression of interferon-beta (IFN-β) and a range of pro-inflammatory factors, which in turn triggers necroptosis—a regulated form of cell death—and amplifies intestinal tissue damage. Functional validation confirms the pathogenic role of cGAS-STING in this process: genetic deletion of STING significantly blocks mtDNA-mediated necroptotic programming and alleviates IIRI (Zhang et al., 2020). A growing body of research has identified pharmacological agents that target the cGAS-STING pathway to mitigate IIRI, with distinct regulatory mechanisms.

For instance, naringin directly protected against IIRI by inhibiting STING phosphorylation, macrophage infiltration, inflammatory signaling, and oxidative injury (Gu et al., 2023). By contrast, evidence for ginsenoside Rg3 is indirect: in an enteric viral infection model, Rg3 remodeled the intestinal microbiota and engaged a GPR43–cGAS–STING/type I interferon axis (Wang G. et al., 2023). This finding supports a plausible microbiota–immune mechanism but should not be interpreted as direct proof of efficacy in vascular IIRI.

#### 2.2.5 Nrf2/HO-1 pathway activators

Nuclear factor erythroid 2-related factor 2 (Nrf2) is a master transcription factor that coordinates the endogenous antioxidant defense program. Under basal conditions, Nrf2 is retained in the cytoplasm by Kelch-like ECH-associated protein 1 (Keap1), which promotes its ubiquitination and proteasomal degradation. Under oxidative or inflammatory stress, Nrf2 dissociates from Keap1, translocates to the nucleus, and binds antioxidant response elements (AREs), thereby initiating transcription of cytoprotective genes such as HO-1 (Ngo and Duennwald, 2022). Because HO-1 generates antioxidant and anti-inflammatory metabolites during heme degradation, the Nrf2/HO-1 axis has become a major focus in experimental IIRI. This pathway has also been implicated in extra-intestinal ischemia-reperfusion settings, including myocardial injury, underscoring a conserved organ-protective role across tissues (Liu C. X. et al., 2024). Preclinical studies suggest that activators of this pathway may preserve mucosal architecture, reduce epithelial permeability, suppress ferroptosis, and dampen inflammatory injury, making Nrf2/HO-1 one of the most consistently reproduced protective programs in the current literature.

For example, Fan et al. (2024) showed that methane-rich saline preserves intestinal mucosal structural integrity, reduces epithelial permeability, improves local microcirculation, decreases ROS generation, and suppresses ferroptosis through modulation of the Nrf2/HO-1 pathway. Similarly, bryostatin-1 attenuates IIRI by activating Nrf2/HO-1 signaling, thereby reducing oxidative stress and inflammatory injury while partially preserving barrier function (Liu et al., 2023). These studies emphasize that the translational value of Nrf2/HO-1 activation lies not only in antioxidant defense itself but also in its downstream effects on barrier maintenance and inflammatory containment. Dimethyl fumarate (DMF), for instance, exerts antioxidant and anti-apoptotic effects by upregulating the Nrf2/HO-1/Bcl-2 axis (Gendy et al., 2021). At the same time, DMF reduces lipid peroxidation, iNOS, MPO, and caspase-3 activity and downregulates inflammatory mediators including TNF-α, NF-κB, IL-1β, and P-selectin. It may also modulate the canonical Wnt/β-catenin pathway through inhibition of GSK-3β, suggesting that some Nrf2/HO-1 activators act through broader cytoprotective networks rather than a single linear axis. This pleiotropic profile is particularly relevant to IIRI, where oxidative stress, inflammation, and cell death are tightly interwoven.

### 2.3 Pharmacological modulation of dysregulated cell death

Ferroptosis is a regulated form of cell death driven by iron-dependent lipid peroxidation and is mechanistically distinct from apoptosis and necrosis. In IIRI, labile iron and reperfusion-associated ROS promote phospholipid peroxidation, membrane disruption, mitochondrial dysfunction, and mucosal injury. Radical-trapping antioxidants can terminate lipid-peroxidation chain reactions and may therefore complement interventions that preserve the GSH/GPX4 axis (Bertoni et al., 2018).

More recently, Feng et al. (2022) demonstrated that apigenin-7-O-β-D-(-6″-p-coumaroyl)-glucopyranoside (APG) attenuates IIRI-induced injury by suppressing ferroptosis. Specifically, APG reduced ROS generation and Fe^2+^ accumulation in injured intestinal tissues, an effect associated with downregulation of HO-1 and MAO-B. Furthermore, APG preserved mitochondrial structural and functional integrity, thereby further inhibiting ferroptosis and exerting a potent protective effect against IIRI.

The mammalian target of rapamycin (mTOR) is a conserved serine/threonine kinase that serves as a central regulator of cell proliferation, survival, and metabolism. Accumulating evidence indicates that mTOR signaling is involved in ischemic injury, highlighting its relevance as a therapeutic target in ischemic disorders (Chong et al., 2013). Rapamycin, a specific inhibitor of mTOR, modulates autophagic activity by suppressing this pathway. In preclinical models relevant to intestinal injury, rapamycin treatment has been shown to significantly increase the LC3-II/LC3-I ratio, a widely used marker of autophagosome formation (Xu Y. et al., 2024). By promoting autophagosome biogenesis, rapamycin facilitates the clearance of damaged organelles and cytotoxic aggregates, thereby mitigating oxidative stress-induced mucosal injury. Additional intestinal studies have further suggested that rapamycin may promote barrier repair through the mTOR/PBLD/AMOT signaling pathway (Xu Y. et al., 2024). Beyond its regulatory role in autophagy, rapamycin also attenuates inflammatory responses by inhibiting NF-κB activation and reducing downstream cytokines such as TNF-α and IL-6 (Xu Y. et al., 2024). Collectively, these findings suggest that rapamycin may serve as a multifaceted therapeutic agent for IIRI through coordinated modulation of autophagy and inflammation. However, much of the cited barrier-repair evidence derives from adjacent intestinal inflammatory models; direct efficacy, dosing, and safety in standardized IIRI models remain to be established.

## 3 Novel targeted drug-delivery systems

Novel targeted delivery systems are currently under development to enable precise drug delivery in response to elevated reactive oxygen species (ROS) levels within inflamed regions of the gastrointestinal tract. The interaction between nanomaterials and Nrf2-related antioxidant responses has attracted growing attention in oxidative-stress-mediated diseases, providing a conceptual basis for these platforms in intestinal injury (Thiruvengadam et al., 2023). Accumulating evidence has demonstrated that ROS-responsive nanoparticles can not only mimic the catalytic activities of antioxidant enzymes—including superoxide dismutase (SOD) and glutathione peroxidase (GPx)—but also efficiently neutralize excessive ROS, thereby alleviating oxidative stress-induced tissue damage. In the context of inflammatory bowel disease (IBD), for instance, ROS-responsive nanoparticles have been employed for the targeted delivery of antioxidant drugs, leading to significantly enhanced therapeutic efficacy (Wan et al., 2025). Beyond IBD, this delivery platform also exhibits considerable potential for IIRI. By facilitating the site-specific delivery of antioxidants such as melatonin and curcumin, it may enhance antioxidant and anti-inflammatory effects while minimizing intestinal tissue destruction. Notably, recent investigations have highlighted the utility of pH-sensitive nanomicelles loaded with resveratrol; these nanocarriers can specifically target ischemic intestinal segments, resulting in a 4.7-fold increase in the bioavailability of resveratrol (Xu J. et al., 2024). In a separate study, Sheng et al. (2025) reported that manganese dioxide nanoparticles coated with macrophage membranes and loaded with rapamycin effectively mitigated IIRI. The underlying mechanism was attributed to the nanoparticles’ ability to reduce oxidative stress and promote autophagy in affected tissues. Additionally, Zhao et al. demonstrated that nanoparticles encapsulating apigenin-7-glucoside exerted a protective effect against IIRI, further validating the promise of ROS-responsive nanosystems (Zhao et al., 2024). Exosomes are a class of extracellular vesicles (EVs) with diameters ranging from 30 nm to 150 nm, which are extensively involved in intercellular communication, disease diagnosis, and targeted therapeutic interventions (Alzhrani et al., 2021). As endogenous mediators of cell-to-cell signaling, exosomes can encapsulate and deliver a diverse array of biomolecules—including microRNAs (miRNAs), circular RNAs (circRNAs), and proteins—to exert protective effects against IIRI.

Notably, porcine milk-derived exosomal miRNAs (e.g., miR-4334, miR-219, and miR-338) have been reported to mitigate lipopolysaccharide (LPS)-induced intestinal epithelial cell damage. The underlying mechanism involves inhibition of cellular apoptosis and inflammatory responses through the p53 and Toll-like receptor 4/nuclear factor-κB (TLR4/NF-κB) signaling pathways (Xie et al., 2019). In the context of IIRI, the expression level of circEZH2_005 in exosomes is significantly downregulated, and this reduction is negatively correlated with the severity of intestinal tissue damage. Functional studies have demonstrated that exosomes overexpressing circEZH2_005 can promote the proliferation of intestinal crypt stem cells and facilitate intestinal mucosal repair. Mechanistically, circEZH2_005 is highly expressed in intestinal crypt cells; its upregulation enables direct interaction with heterogeneous nuclear ribonucleoprotein A1 (hnRNPA1), thereby promoting the proliferation of leucine-rich repeat-containing G-protein coupled receptor five-positive (Lgr5^+^) stem cells and enhancing G protein-coupled receptor class C group 5 member A (*Gprc5a*) stability. Collectively, these effects contribute to the attenuation of IIRI-associated intestinal mucosal injury (Zhang et al., 2023). Thus, exosomal circEZH2_005 holds promise as a potential biomarker for IIRI, and targeting the circEZH2_005/hnRNPA1/*Gprc5a* axis may represent a novel therapeutic strategy for this condition. Beyond their intrinsic regulatory roles, exosomes also serve as versatile drug-delivery carriers for targeted delivery of antioxidants and anti-inflammatory agents such as melatonin and curcumin to damaged intestinal tissues, thereby enhancing therapeutic efficacy. For instance, Zhang et al. showed that exosomes derived from bone marrow mesenchymal stem cells (BMSCs) and loaded with miR-144-3p ameliorated IIRI by modulating oxidative stress and regulating the phosphatase and tensin homolog (PTEN)/protein kinase B (Akt)/nuclear factor E2-related factor 2 (Nrf2) pathway (Zhang et al., 2022). Additionally, these exosomes were found to regulate immune responses and reduce neuronal apoptosis through the TLR4/NF-κB pathway (Zhang et al., 2022; Sheng et al., 2021). Conversely, gut-derived exosomes may also exert pathogenic effects during IIRI, as they have been shown to induce liver damage by promoting the polarization of M1 macrophages in the liver. Consequently, inhibiting the secretion of gut-derived exosomes has emerged as a potential therapeutic target for preventing post-I/R liver dysfunction and memory impairment. In support of this, Wang et al. demonstrated that exosomes isolated from human breast milk alleviated intestinal damage in I/R-induced rat models by suppressing intestinal hyperplasia and reducing the expression of the pro-inflammatory cytokine tumor necrosis factor-α (TNF-α) (Wang et al., 2022).

Recent studies have further strengthened the case for exosome-based intestinal delivery. Chen et al. developed milk-derived exosomes loaded with siRNA targeting CCL7 (M-Exo/siCCL7) and showed that this engineered platform attenuated murine IIRI, as evidenced by reduced histological damage, lower serum markers of barrier dysfunction, and decreased systemic inflammation, thereby extending exosome utility from biomarker transport to actively engineered nucleic-acid therapy (Chen et al., 2025). Additional 2025 studies indicate that exosome engineering may also expand mechanistic reach. Resveratrol-primed adipose stem cell-derived exosomes were reported to alleviate IIRI by regulating macrophage polarization and suppressing mucosal immune dysregulation (Ye et al., 2025), while bone-marrow-mesenchymal-stem-cell-derived extracellular vesicles delivering miR-378a-3p inhibited ferroptosis through the SREBF2/HMGB1 axis in intestinal I/R models (Liu Z. et al., 2025). Together, these findings suggest that next-generation exosome systems may be developed not only as passive carriers but also as programmable platforms for anti-inflammatory, immunoregulatory, and ferroptosis-targeted intervention.

Direct evidence for conventional liposomes or lipid nanoparticles in IIRI remains limited. Macrophage-membrane-coated MnO_2_ nanoparticles loaded with rapamycin are not conventional liposomes, but they illustrate how a biologically derived membrane interface can improve lesion targeting while reducing ROS, enhancing autophagy, and suppressing inflammatory cytokines in murine IIRI (Sheng et al., 2025). Evidence from intestinal inflammatory disease and myocardial ischemia–reperfusion models further suggests that lipid-based nanocarriers can improve cargo stability and lesion-site exposure (Zhu et al., 2025; Shi et al., 2025), but these findings remain indirect for IIRI.

Accordingly, proposed advantages of lipid-based carriers in IIRI—including ROS-responsive release, improved mucosal penetration, and reduced systemic exposure—remain hypotheses requiring direct pharmacokinetic, biodistribution, efficacy, and safety testing in standardized IIRI models. Extrapolation from inflammatory bowel disease or myocardial ischemia–reperfusion should be clearly distinguished from intestine-specific evidence (Zhu et al., 2025; Shi et al., 2025).

## 4 Translational barriers and comparative therapeutic potential

Several factors may account for this persistent translational gap. First, experimental IIRI models are biologically simplified and usually involve brief, controlled superior mesenteric artery occlusion in otherwise healthy animals, whereas human intestinal ischemia often occurs in the context of shock, thrombosis, infection, advanced age, vascular disease, or multiple-organ dysfunction. Second, many candidate agents are administered before ischemia or immediately before reperfusion in preclinical studies, but such tightly controlled time windows are difficult to reproduce in emergency and peri-operative practice. Third, clinical studies must prioritize hard outcomes and safety, whereas preclinical studies often focus on mechanistic readouts that are necessary but not sufficient for real therapeutic adoption. These differences help explain why clinically meaningful pharmacological trials remain scarce and why positive experimental findings have been difficult to translate into routine practice.

A comparative view of translational promise also helps contextualize the current evidence base. Agents such as NAC, melatonin, metformin, and dexmedetomidine presently appear stronger in terms of reproducibility because protective effects have been reported across more than one experimental context and span biochemical, histologic, barrier-related, and remote-organ readouts. By contrast, highly pathway-focused interventions such as naringin, bryostatin-1, or APG provide important mechanistic precision but still rely on a more limited replication base. Timing flexibility is another critical differentiator: most candidate therapies are evaluated as pretreatments, whereas regimens that remain active around or after reperfusion may be more clinically realistic. In this regard, cromolyn sodium has shown benefit in reperfusion-oriented treatment paradigms, emphasizing the importance of therapeutic window rather than prophylaxis alone (Hei et al., 2007; Hei et al., 2008). Multi-target agents such as melatonin, metformin, honokiol, and dexmedetomidine may also be more attractive translationally because they integrate antioxidant, anti-inflammatory, barrier-protective, and anti-apoptotic or mitochondrial-stabilizing actions. Finally, clinically familiar agents with established human safety experience—including NAC, melatonin, metformin, dexmedetomidine, heparin, and verapamil—may offer greater near-term feasibility for repurposing than newly engineered compounds, even when the latter provide greater mechanistic specificity in experimental systems.

## 5 Future perspectives and limitations

Future research on IIRI should continue to deepen mechanistic understanding while maintaining a clear therapeutic orientation. Because oxidative stress, inflammatory amplification, barrier dysfunction, microcirculatory impairment, and dysregulated cell death are tightly interconnected, multi-target intervention may be more realistic than strict single-pathway inhibition. In this context, agents with pleiotropic effects and rational drug combinations deserve particular attention.

Combination therapy warrants particular attention because IIRI is not organized around a single dominant pathway. NAC plus the platelet-activating-factor inhibitor lexipafant protected rats against intestinal IIRI, although the effect was not explained solely by reduced ICAM-1 expression (Olanders et al., 2003). A COX-2 inhibitor combined with antioxidant therapy broadened suppression of oxidative and inflammatory injury (Gugliandolo et al., 2020). In a 2025 rat acute mesenteric ischemia model, heparin plus verapamil administered at revascularization also mitigated intestinal injury without obvious adverse systemic effects (Wilken et al., 2025). Immune-directed combinations remain earlier in development: combined PD-1 and IL-10 blockade was reported to reinvigorate mucosal CD8^+^ T-cell responses and reduce bacterial translocation and liver injury after IIRI (Liu Z. M. et al., 2025). Collectively, these findings support multimodal regimens that couple barrier protection with control of oxidative stress, inflammation, microcirculatory dysfunction, and cell-death signaling, but comparative efficacy and safety studies are still required.

Advanced delivery systems are also likely to become increasingly important. ROS-responsive nanoparticles, exosome-based carriers, and liposomal platforms may improve local drug exposure, protect unstable cargos, and reduce systemic toxicity, thereby enhancing the therapeutic performance of candidate agents that otherwise show limited intestinal penetration or narrow therapeutic windows.

In addition, future preclinical studies should place greater emphasis on clinically relevant end points, including bowel viability, barrier recovery, remote organ protection, and survival, rather than relying solely on histology or oxidative-stress biomarkers. Biomarker-guided patient stratification may also help identify subgroups most likely to benefit from intervention, especially in peri-operative and critical-care settings where occult intestinal hypoperfusion is often underrecognized.

Several limitations should be acknowledged. The available evidence is generally of low-to-moderate quality and is derived mainly from single-center animal studies, prophylactic designs, heterogeneous protocols, and limited independent replication. Differences in species, ischemia and reperfusion durations, dose, treatment timing, and outcome definitions constrain direct comparisons. Some pathway and delivery-system evidence is extrapolated from adjacent intestinal inflammatory or extra-intestinal ischemia–reperfusion models. Standardized preclinical protocols and reporting, together with direct comparative studies, are therefore needed.

Overall, the field remains promising because a large body of mechanistic evidence has already identified multiple actionable targets and several candidate drugs with reproducible protective effects in experimental models. The next stage of development should focus on better integrating pharmacological targeting, delivery optimization, and clinically realistic study design so as to improve the feasibility of future bedside translation.
